# Rosmarinic acid, the active component of Rubi Fructus, induces apoptosis of SGC-7901 and HepG2 cells through mitochondrial pathway and exerts anti-tumor effect

**DOI:** 10.1007/s00210-023-02552-z

**Published:** 2023-06-20

**Authors:** Changlun Chen, Yilin Liu, Yi Shen, Lili Zhu, Lumeng Yao, Xingxing Wang, Anna Zhang, Jiao Li, Jianjun Wu, Luping Qin

**Affiliations:** College of Pharmaceutical Sciences, Zhejiang Chinese Medical University, Hangzhou, 311402 China

**Keywords:** Rubi Fructus, Rosmarinic acid, Apoptosis, Mitochondria apoptosis pathway, Anti-tumor

## Abstract

Rosmarinic acid (RA) is a well-known phenolic acid widely present in over 160 species of herbal plants and known to exhibit anti-tumor effects on breast, prostate, and colon cancers in vitro. However, its effect and mechanism in gastric cancer and liver cancer are unclear. Moreover, there is no RA report yet in the chemical constituents of Rubi Fructus (RF). In this study, RA was isolated from RF for the first time, and the effect and mechanism of RA on gastric and liver cancers were evaluated using SGC-7901 and HepG2 cells models. The cells were treated with different concentrations of RA (50, 75, and 100 μg/mL) for 48 h, and the effect of RA on cell proliferation was evaluated by the CCK-8 assay. The effect of RA on cell morphology and mobility was observed by inverted fluorescence microscopy, cell apoptosis and cell cycle were determined by flow cytometry, and the expression of apoptosis-related proteins cytochrome C, cleaved caspase-3, Bax, and Bcl-2 was detected by western blotting. The results revealed that, with an increase in the RA concentration, the cell viability, mobility, and Bcl-2 expression decreased, while the apoptosis rate, Bax, cytochrome C, and cleaved caspase-3 expression increased, and SGC-7901 and HepG2 cells could be induced to arrest their cell cycle in the G0/G1 and S phases, respectively. These results together indicate that RA can induce apoptosis of SGC-7901 and HepG2 cells through the mitochondrial pathway. Thus, this study supplements the material basis of the anti-tumor activity of RF and provides an insight into the potential mechanism of RA-inducing apoptosis of gastric cancer SGC-7901 cells and liver cancer HepG2 cells, thereby facilitating further developmental studies on and the utilization of the anti-tumor activity of RF.

**Supplementary information:**

The online version contains supplementary material available at 10.1007/s00210-023-02552-z.

## Introduction

Rosmarinic acid (RA) is a water-soluble natural phenolic acid widely present across 160 species of herbal plants (Guan et al. 2022), originally isolated and named after rosemary (*Rosmarinus officinalis*, Lamiaceae). Existing studies suggest that RA has anti-tumor effect in breast cancer (reducing methyltransferase activity and hypermethylation of DNA) (Paluszczak et al. 2010), prostate cancer (inducing apoptosis) (Yesil-Celiktas et al. 2010), and colon cancer (inhibiting the activity of COX2 protein promoter) (Scheckel et al. 2008). For liver cancer and gastric cancer, RA led to decrease cell viability and morphological changes in HepG2 cells (Renzulli et al. 2004) as well as a decrease in the survival of SGC-7901 cells (Li et al. 2013). However, these studies only demonstrated RA’s anti-tumor effect on gastric and liver cancers, with no establishment of the specific underlying mechanisms. Therefore, we aimed to explore the mechanism RA exerted anti-tumor effect on gastric and liver cancers.

*Rubus chingii* Hu., belonging to the family Rosaceae, whose fruits are known as Rubi Fructus (RF), is widely planted for its significant nutritional and medicinal values. The RF is both medicine and food and has been used as traditional Chinese medicine (TCM) for more than 1500 years. In recent years, 239 active components including flavonoids (Yu et al. 2019; Sheng et al. 2020), terpenoids (Yu et al. 2019; Sheng et al. 2020), alkaloids (Yu et al. 2019; Sheng et al. 2020), steroids (Yu et al. 2019; Sheng et al. 2020), organic acids, and others (Yu et al. 2019; Sheng et al. 2020) isolated from RF, which possess antioxidants (Nan et al. 2013), anti-inflammatory (Nan et al. 2013), antimicrobial (Zhu 2012), anti-osteoporosis (Liang et al. 2015), anti-aging, and anti-tumor (Zeng et al. 2018) activities. Although several reports are available on the biological activity of RF, they are mostly focused on its kidney-tonifying, anti-inflammatory, and antioxidant properties, with very few reports on its anti-tumor effect, and the researches are limited to study of the crude extract of RF (Zhang et al. 2015). RF extract has been proven to exhibit anti-tumor activities on liver and gastric cancers. However, these studies could not identify which precise component of RF plays the major role in this effect. Thus, the mechanism and material basis of RF anti-tumor activity remain unclear.

The global morbidity and mortality rates of cancer as a malignant disease are increasing every year, and this upward trend is more pronounced in the elderly population (Xia et al. 2022). Among them, gastric and liver cancers account for the second and third most cases of cancer deaths worldwide (Frager and Schwartz 2020). Due to the problem of dietary habits, the incidence of gastric cancer and liver cancer is the highest in China (Liu and Song 2021). In our previous study on RF, RA was isolated for the first time in the literature. Given the literature reports, neither the material basis of RF anti-tumor nor the mechanism of RA on SGC-7901 and HepG2 is clear; therefore, we studied the mechanism of RA on these two cell types, with the hope to provide a reference for elucidating the material basis of RF anti-tumor effect and the mechanism on SGC-7901 and HepG2 cells. We believe that our findings will provide a reference for the development and utilization of RF in anti-tumor roles in the future.

## Materials and methods

### Reagent treatment and plant materials

RA was obtained from RF by separation and purification. A stock solution of RA was prepared at a 100 mg/mL concentration in dimethyl sulfoxide (DMSO; Sigma, St. Louis) and stored at 4 °C. After diluting the stock solution 1000 times before each use, the concentration of DMSO was kept at < 0.1%. According to the pre-experimental results, we selected the concentration of cell administration as 50, 75, and 100 μg/mL of RA for the test group.

RF was obtained from Pan’an County, Zhejiang Province, and identified by Professor Luping Qin, College of Pharmaceutical Sciences, Zhejiang Chinese Medical University. The voucher specimen has been deposited at the College of Pharmaceutical Sciences, Zhejiang Chinese Medical University, for future reference.

### Extraction and isolation

RF (10 kg) was crushed and passed through a 100-mesh sieve to obtain a uniform powder, which was then heated and refluxed thrice with 5 L 95% ethanol (3 h each time), followed by a combination with the alcohol extract for 3 times and concentration to a non-alcoholic flavor by using a rotary evaporator to obtain the ethanol extract. The ethanol extract (303 g) was suspended in 1 L water and extracted with the same amount of petroleum ether, dichloromethane, ethyl acetate, and n-butanol.

**Fig. 1:**
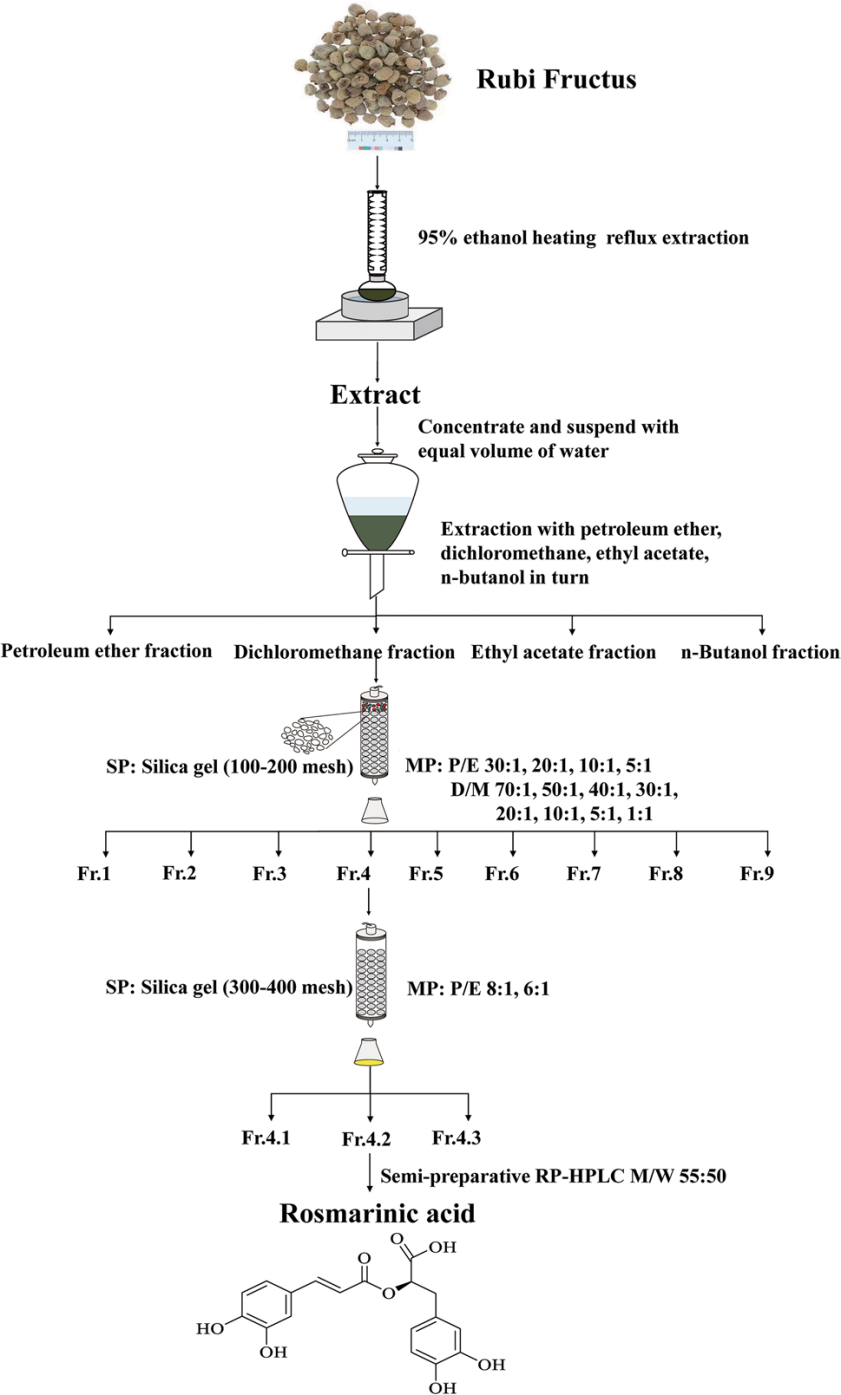
Flow chart of extraction and separation of RA. P, petroleum ether. E, ethyl acetate. D, dichloromethane. M, methyl alcohol. W, ultrapure water. SP, stationary phase. MP, mobile phase

The CH_2_Cl_2_ extract (12 g) was subjected to 100–200 mesh silica gel column using PE-EtOAc (30:1–5:1) and CH_2_Cl_2_-MeOH (70:1–1:1) as the flow-dependent elution to obtain 9 groups of fractions (Fr.1–9). Fr.4 (686.3 mg) was repeatedly eluted on a 300–400-mesh silica gel column by PE-EtOAc (8:1–6:1) to obtain 3 groups of fractions: Fr.4.1, 4.2, and 4.3. Fr.4.2 (261.2 mg) was purified by semi-preparative RP-HPLC applying MeOH-H_2_O (55:50) as the mobile phase to obtain compound **1** (7.1 mg). Flow chart of extraction and separation of rosmarinic acid was shown as Fig. 1.

### Cell culture

The human hepatoma cell line HepG2, gastric cancer cell line SGC-7901, human normal gastric mucosa epithelial cell line GES-1, and human normal lung epithelial cell line BEAS-2B were obtained from the Cell Bank of the Type Culture Collection of the Chinese Academy of Sciences (Shanghai, China). All cells were cultured in Dulbecco’s Modified Eagle’s Medium (DMEM, Gibco) and supplemented with 10% fetal bovine serum (FBS, Gibco), 100 U/mL penicillin, and 100 μg/mL streptomycin (PS, Gibco). All cells were maintained at 37 °C in a humidified atmosphere of 5% CO_2_.

### Cell viability assay

The cell viabilities of HepG2, SGC-7901, GES-1, and BEAS-2B cells were performed by using the Cell Counting Kit-8 Assay (CCK-8; Biosharp). Briefly, the cells were seeded in a 96-well microplate at the density of 3 × 10^3^ cells/well. After 24 h, the RA-containing stock solutions were serially diluted to dosing concentrations of 100, 75, and 50 μg/mL in a DMEM medium and added to the designated wells. After incubation for 48 h, the DMEM medium was replaced with 10 uL of the CCK-8 solution and after further incubation for 1 h at 37 °C in the dark. The absorbance of each well was measured at 450 nm by using a microplate reader (Thermo Fisher Scientific, Inc.).

### Cell morphological observations

The experimental cells were seeded in a 6-well plate at the density of 3 × 10^5^ cells/well. After 24 h, the original medium in the 6-well plate was aspirated, and a serum-free medium containing different concentrations of RA was added. After 48 h of incubation, the cell morphology changes were observed, and the images were captured using an inverted fluorescence microscope (Nikon-Tls, Japan). The staining operation was carried out according to the instructions of Hoechst 33,258 kit, and the apoptotic cells were observed and photographed under inverted fluorescence microscope.

### Cell migration assay

The cell migration ability was assessed according to the wound-healing assays described in the literature (Zhang et al. 2018). HepG2 and SGC-7901 cells were seeded in a 6-well plate at the density of 3 × 10^5^ well. After 24 h, discard the original medium, using a 200-μL pipette tip, and 3 parallel vertical scratches were made on the cell monolayer in each well. Subsequently, cells were cultured in serum-free medium containing different concentrations of RA. At 0 and 24 h after administration, the cells were photographed using an inverted fluorescence microscope to record the changes in the scratched area, and the wound area was calculated by the Image J software. The expression level of cell migration protein MMP9 was detected by western blot.

### Apoptosis analysis

Logarithmic growth-phase HepG2 and SGC-7901 cells were seeded in a 6-well plate at the density of 3 × 10^5^ cells/well and cultured overnight. DMEM medium containing different concentrations of RA was added to continue the culture for 48 h. Next, apoptosis was detected using the Annexin V-FITC Apoptosis Detection Kit (Beyotime Institute of Biotechnology). Briefly, floating and adherent cells in the plate were collected and washed twice with pre-cooled PBS. Then, 195 μL of annexin V-FITC buffer was used to resuspend the cells, to which 5 μL of annexin V-FITC staining solution and 10 μL of PI staining solution was added, mixed well, and incubated at room temperature for 30 min in the dark. The cell apoptosis was analyzed by the CytoFlex Flow Cytometer (Beckman Coulter, Inc., USA).

### Cell cycle analysis

The cell cycle distribution of HepG2 and SGC-7901 cells was evaluated by flow cytometry using the Cell Cycle and Apoptosis Analysis Kit (Beyotime Institute of Biotechnology). As described in the literature (Sablowski and Carnier Dornelas 2014), the cells were seeded in a 6-well plate at the density of 3 × 10^5^ cells/well and cultured overnight. Then, a culture medium containing different concentrations of RA was added to the cells and incubated for 48 h. Subsequently, the collected cells were placed in a centrifuge tube, fixed with 75% ethanol, and placed in 4 °C overnight. Next, the configured PI/RNase staining buffer was added to the centrifuge tube and incubated for 30 min in the dark at room temperature. The cell cycle was analyzed by the CytoFlex flow cytometer. The DNA content at G0/G1, S, and G2/M phases was analyzed by using the FlowJo software.

### Western blotting

HepG2 and SGC-7901 cells were seeded in a 6-well plate at the density of 3 × 10^5^ cells/well and treated with a media containing different concentrations of RA for 48 h and collecting cells. Briefly, the quantified proteins were separated by SDS–polyacrylamide gel electrophoresis; subsequently, the protein was transferred onto a PVDF membrane. The PVDF membranes were then blocked with a blocking solution for 2 h and incubated with the following primary antibodies overnight at 4 °C: anti-Bcl-2, anti-Bax, anti-caspase-3, and anti-cytochrome C (all from Cell Signaling Technology). The PVDF membrane was washed three with the Western special washing solution for 10 min each time. Then, the membrane was incubated in horseradish peroxidase-conjugated IgG secondary antibody (Cell Signaling Technology) for 1 h at room temperature. Finally, the membrane was developed using the BeyoECL Star Kit, and the proteins were visualized with a chemiluminescence system. The protein bands were processed by ImageJ software (National Institutes of Health), and β-actin (Cell Signaling Technology) was used as an internal control amount to quantify the Western blotting bands.

### Statistical analyses

Data were expressed as the mean ± SD. All experiments were independently replicated at least thrice. Statistical analysis was performed by using the GraphPad Prism 9 software. Nonparametric data were compared using Mann–Whitney *U*-test, and the parametric data were compared using Student’s *t*-test. A statistically significant difference was considered acceptable at *P* < 0.05.

## Results

### Isolation of RA from Rubi Fructus

**Fig. 2:**
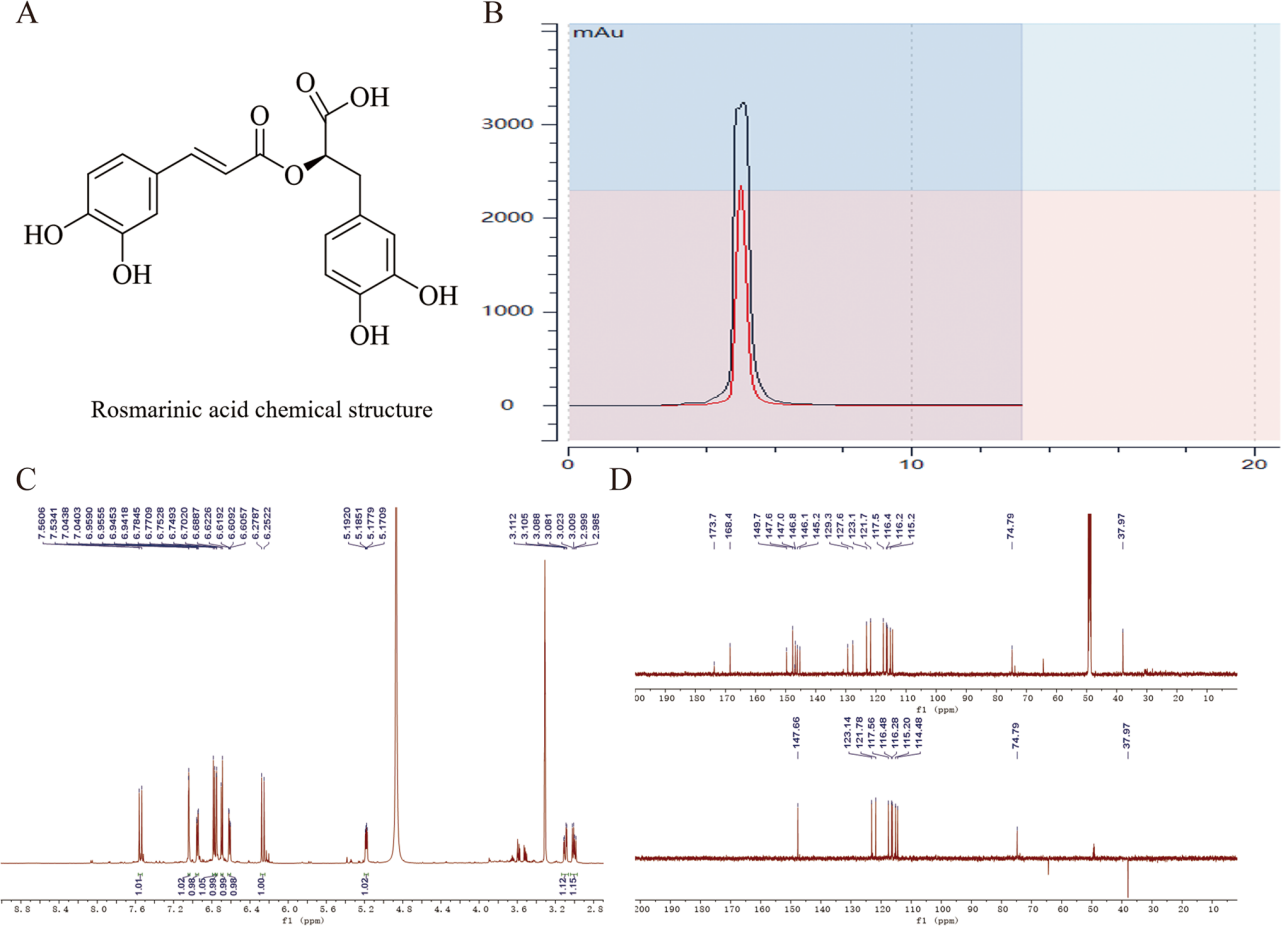
Nuclear magnetic resonance and purity identification of rosmarinic acid. **A** Rosmarinic acid chemical structure. **B** Liquid phase diagram of purity identification. The mobile phase condition was Acetonitrile-H_2_O (54–46), the detection wavelength was 254 nm and 210 nm, and the purity of RA was more than 97%. **C **^1^H NMR. **D**.^13^C NMR and DEPT(135°)

RA isolated from RF for the first time. The results of NMR identification confirmed the obtained compound was RA (Kang and Lee 2011), and its structure is shown in Fig. 1. It was a polyphenolic natural phenolic acid compound, light-yellow powder, and easily soluble in water, ethanol. The NMR data are as follows: ^1^H NMR (600 MHz, CD_3_OD) δ 7.55 (d, *J* = 15.9 Hz, 1H, H-7), 7.04 (d, *J* = 2.1 Hz, 1H, H-2), 6.95 (dd, *J* = 8.2, 2.1 Hz, 1H, H-6), 6.78 (d, *J* = 8.1 Hz, 1H, H-5’), 6.61 (dd, *J* = 8.1, 2.1 Hz, 1H, H-5), 6.27 (d, *J* = 15.9 Hz, 1H, H-8), 5.18 (dd, *J* = 8.5, 4.2 Hz, 1H, H-8’), 3.10 (dd, *J* = 14.3, 4.2 Hz, 1H, H-7’ α), 3.00 (dd, *J* = 14.4, 8.5 Hz, 1H, H-7’β). ^13^C NMR (151 MHz, CD_3_OD) δ 173.75 (C-9’), 168.48 (C-9), 149.72 (C-4’), 147.66 (C-7), 146.81 (C-3’), 146.15 (C-4), 145.25 (C-3), 129.37 (C-1’), 127.67 (C-1), 123.14 (C-6), 121.78 (C-5), 117.56 (C-2’), 116.48 (C-5’), 116.28 (C-6’), 115.20 (C-2), 114.18 (C-8),74.79 (C-8’), 37.97 (C-7’). Its ^1^H NMR and ^13^C NMR and the liquid phase diagram for purity identification were shown in Fig. 2 and two-dimensional NMR data (COSY, HSQC, and HMBC were shown as Fig. S1 in Supplementary Material).

### The effect of RA on the viability of HepG2 and SGC-7901 cells

**Fig. 3:**
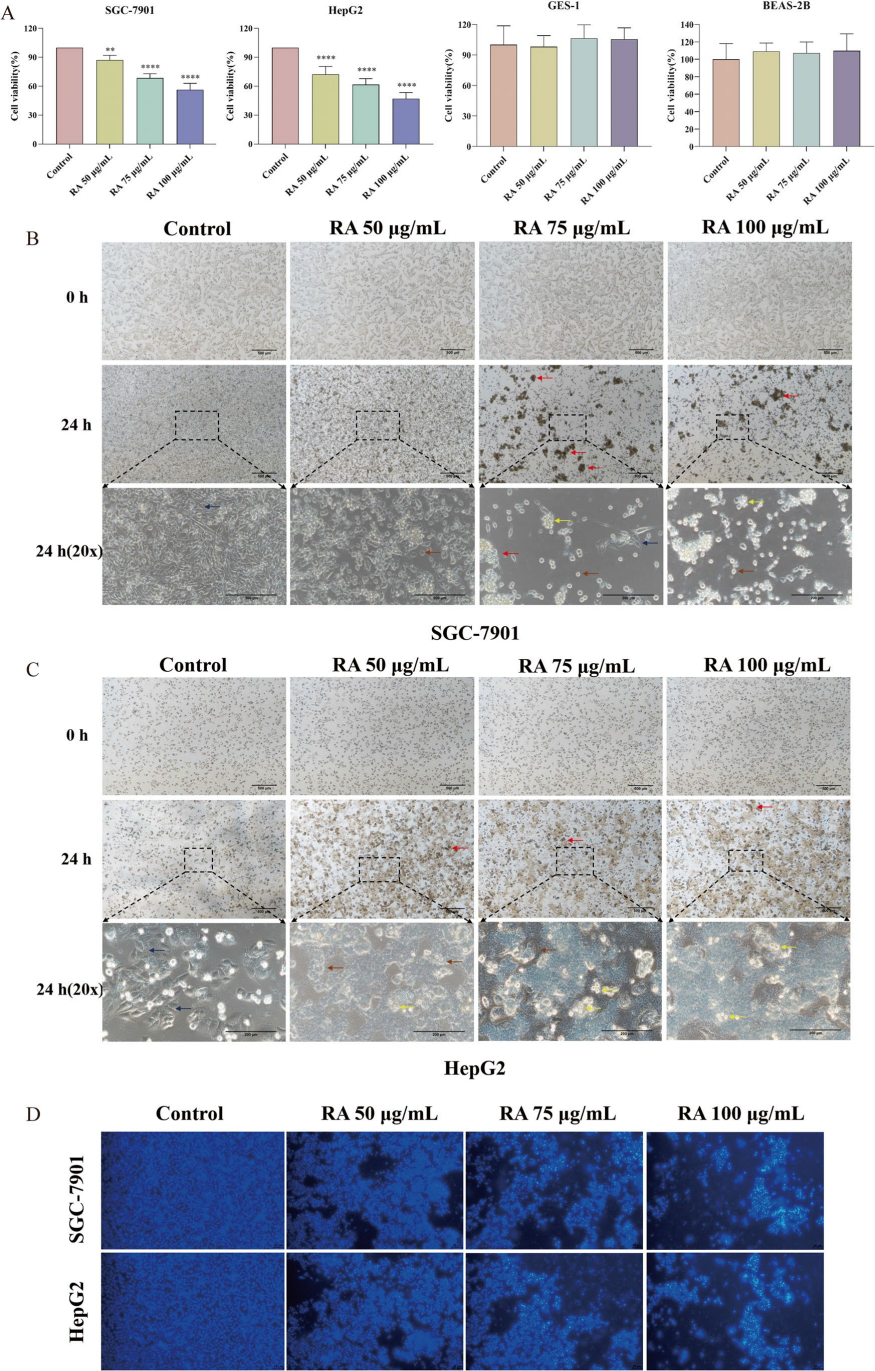
The effect of RA on the viability of HepG2, SGC-7901 cells. **A** SGC-7901 cell viability (*n* = 5); HepG2 cell viability (*n* = 5). **B** SGC cell morphology. **C** HepG2 cell morphology. Red arrows indicate floating dead cells, blue arrows indicate normal cells, brown arrows indicate morphologically altered cells, and yellow arrows indicate apoptotic bodies. **D** Apoptotic cells were observed by Hoechst 33,258 staining. The apoptotic cells in the picture are white and shiny. Magnification factor 40 × . The data are presented as the mean ± SD from five independent experiments, *P < 0.05, **P < 0.01, ***P < 0.001 vs control. Cells not treated were used as control

To evaluate the effect of RA on the viability of HepG2 and SGC-7901 cells, the CCK-8 assay was used to detect the proliferation of HepG2 and SGC-7901 cells. As shown in Fig. 3A, RA significantly decreased the proliferation of HepG2 (IC_50_ = 105.4 μg/mL, 72.34%, 61.77%, 47.11%) and SGC-7901 (IC_50_ = 109.9 μg/mL, 87.20%, 68.53%, 56.35%) in a dose-dependent manner. For GES-1 and BEAS-2B cells, RA had no effect on cell viability and growth. To confirm the results of the CCK-8 assay, we recorded the effect of different concentrations of RA on cell morphology through inverted fluorescence microscopy. After RA treatment, the density of SGC-7901 and HepG2 cells decreased with increasing concentrations of RA, and both the cells exhibited specific apoptotic morphological changes, including cell volume reduction, cell membrane disruption, and apoptosis, thereby the appearance of apoptotic bodies (Fig. 3B and C). As the concentration of RA increases, both the number of floating dead cells and the cell debris scattered at the bottom of the petri dish tended to increase. On the other hand, by magnifying the photo at 20 × , it was observed that the shape of the nucleus also became irregular, and the chromatin became concentrated and even broken. As shown in Fig. 3D, apoptotic cells were observed by Hoechst 33,258 staining. The number of apoptotic cells in HepG2 and SGC-7901 groups increased with the increase of dose, and the number of apoptotic cells in the maximum concentration 100 μg/mL group was the highest compared with the control group. The results of these cell morphologies were consistent with those of the CCK-8 assay, both of which indicated that RA had different degrees of inhibitory effect on the growth of two types of cells and induced apoptosis.

### RA inhibits SGC-7901 and HepG2 cell migration

**Fig. 4:**
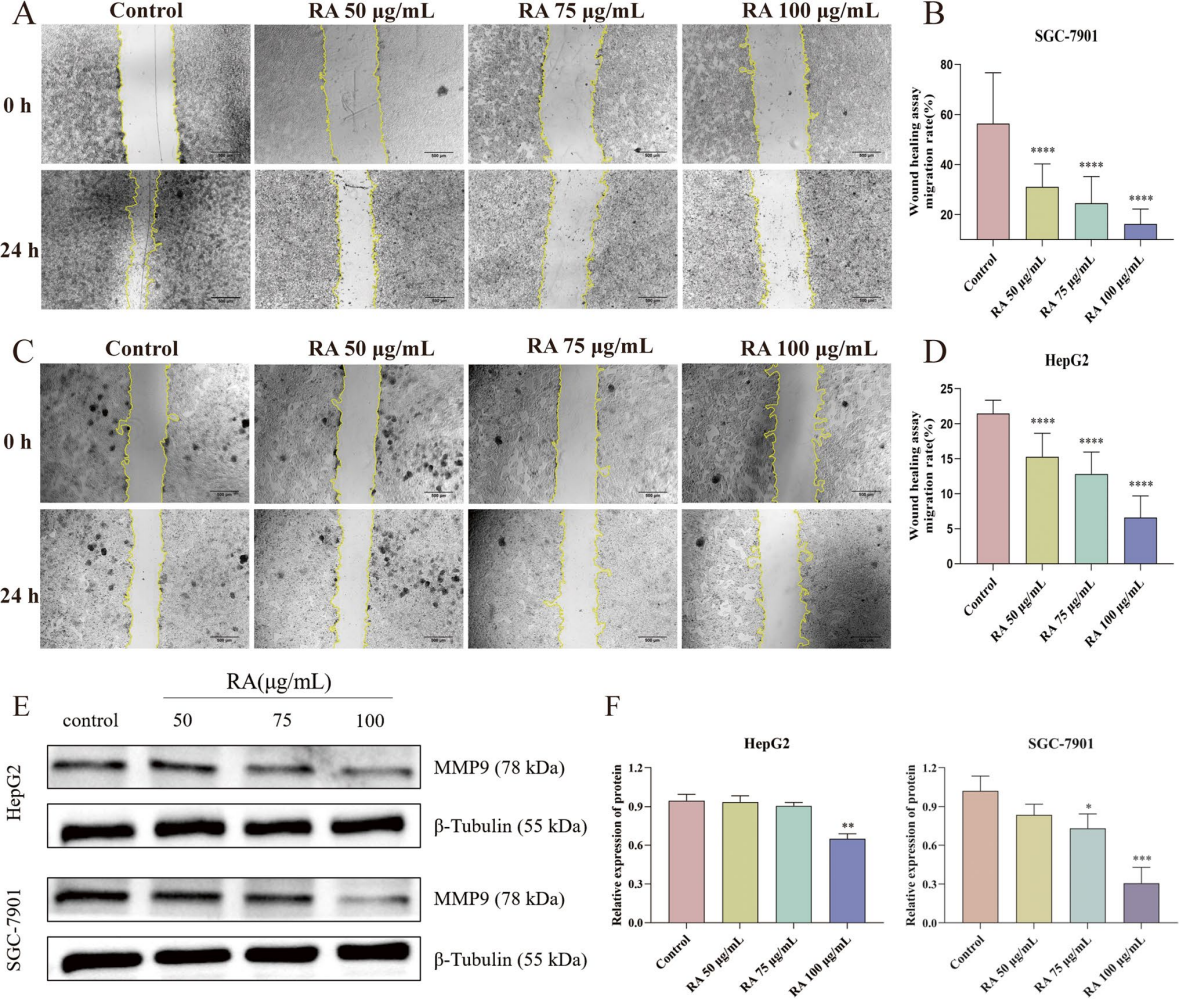
RA inhibits SGC-7901 and HepG2 cell migration. **A** Effect of different concentrations of RA on SGC cell migration. **B** Inhibition rate of migration of SGC cells by different concentrations of RA (*n* = 9). **C** Effect of different concentrations of RA on HepG2 cell migration.** D** Inhibition rate of migration of HepG2 cells by different concentrations of RA (*n* = 9). **E** SGC-7901 and HepG2 cell migration-related protein band. **F** Western blot analysis of protein expressions of MMP9. The data are presented as the mean ± SD from nine independent experiments, *P < 0.05, **P < 0.01, ***P < 0.001 vs control. Cells not treated were used as control

The inhibitory effect of RA on SGC-7901 and HepG2 cell migration was assessed by scratch healing assay. After RA treatment 24 h, both the cell migrations were significantly inhibited (Fig. 4A and C). Specifically, RA inhibited the migration of SGC-7901 cells better than that of HepG2 cells. In SGC-7901 cells, the wound-healing rate of the control group reached 56.38% after 24 h, while that of the groups treated with 50, 75, and 100 μg/mL RA were only 31.01%, 24.50%, and 16.20% (Fig. 4B). In HepG2 cells, the 24 h wound-healing rate in the control group was only 21.44%, while in the groups administered with different concentrations of RA, it was 15.31%, 12.85%, and 6.59%, respectively (Fig. 4D). However, the inhibitory effects of different concentrations of RA on the migration of both the cell types were dose-dependent (Fig. 4B and D), showing a tendency for the higher the dose, the stronger the inhibitory effect. In addition, western blotting was used to detect the expression of migration protein MMP9 after administration. As shown in Fig. 4E and F, the expression of MMP9 protein in HepG2 and SGC-7901 showed a decreasing trend. The higher the concentration of RA, the lower the expression of MMP9 protein, indicating that the increase of RA concentration can significantly inhibit the expression of cell migration protein MMP9, thus inhibiting cell migration. These data suggested that RA possesses the inhibitory activity of SGC-7901 and HepG2 cell migration and that its inhibitory effect on the migration of SGC-7901 cells is stronger than that of HepG2 cells.

### RA promotes apoptosis in SGC-7901 and HepG2 cells

**Fig. 5:**
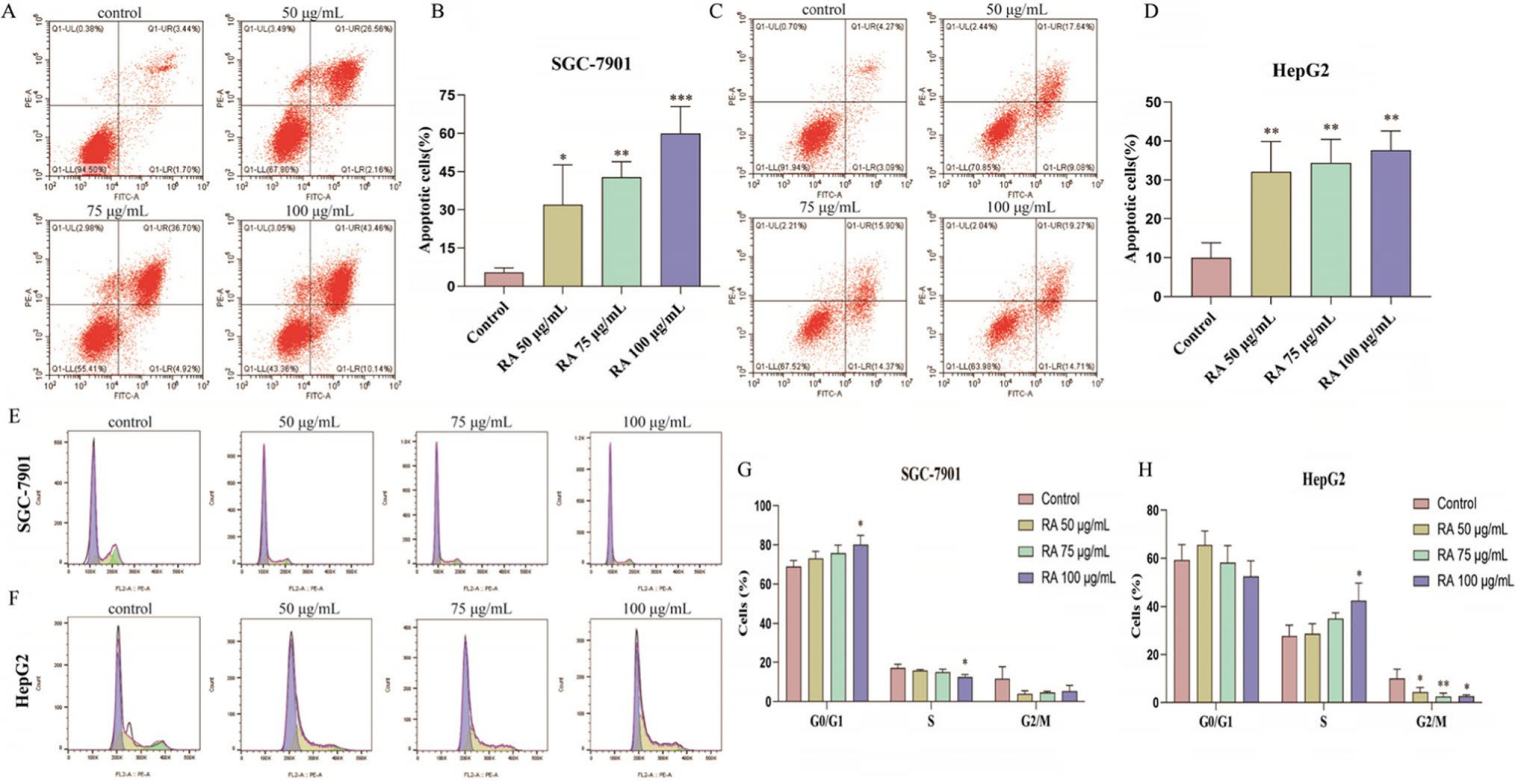
RA promotes apoptosis and induces cell cycle arrest in SGC-7901 and HepG2 cells. **A** Effect of different concentrations of RA on apoptosis of SGC cells. **B** Apoptosis rate of SGC cells in different concentrations of RA (*n* = 3). **C** Effect of different concentrations of RA on apoptosis of HepG2 cells. **D** Apoptosis rate of HepG2 cells in different concentrations of RA (*n* = 3). **E** Effect of different concentrations of RA on cell cycle of SGC. **F** Effect of different concentrations of RA on cell cycle of HepG2. **G** Proportion of SGC cells in different phases after treatment with RA of different concentrations (*n* = 3). **H** Proportion of HepG2 cells in different phases after treatment with RA of different concentrations (*n* = 3). The data are presented as the mean ± SD from three independent experiments, *P < 0.05, **P < 0.01, ***P < 0.001 vs control. Cells not treated were used as control

To further verify the effect of RA on apoptosis induction of SGC-7901 and HepG2 cells, the cells were double-stained by annexin V-FITC/PI kit, after which the apoptosis rate was detected by flow cytometry. As shown in Fig. 5A and C, the apoptosis rate of SGC-7901 and HepG2 cells treated with RA showed an upward trend relative to the control group. The overall apoptosis rate of SGC-7901 cells was higher than that of HepG2 cells, indicating that RA had a better effect on the induction of apoptosis of SGC-7901 cells. On the other hand, the late apoptosis rate of SGC-7901 cells also increased with an increase in the RA concentration, and the ratios of 3.44%, 26.56%, 36.70%, and 43.46%, respectively, while the late apoptosis of HepG2 cells treated with 75 and 100 μg/mL concentration of RA was almost the same. Overall, the rate of apoptosis in the two groups was correlated with the RA concentration in a dose-dependent manner (Fig. 5B and D). Finally, the cumulative data further suggests that RA could induce the apoptosis in SGC-7901 and HepG2 cells.

### RA induces cell cycle arrest in SGC-7901 and HepG2 cells

To explore whether the inhibitory effect of RA on SGC-7901 and HepG2 cells viability was related to its induction of cell cycle arrest, the two cells were treated with RA and detected by flow cytometry. After treated with RA, compared with the control group, the ratio of the G0/G1 phase increased in a dose-dependent manner, such that the S and G2/M phase ratio in the SGC-7901 cells decreased (Fig. 5E and G). In HepG2 cells, the proportions of each period are depicted in Fig. 5F. Compared with the control group, the proportion of the S phase increased, while that of the G2/M phase showed a significant downward trend. The ratio of HepG2 cells in the S phase to G2/M phase distribution was also dose-dependent on the concentration of RA (Fig. 5H). Overall, RA could induce the cell cycle arrest in the SGC-7901 and HepG2 cells, thereby reducing their respective cell viability.

### RA promotes apoptosis by activating the caspase-3 signaling pathways

**Fig. 6:**
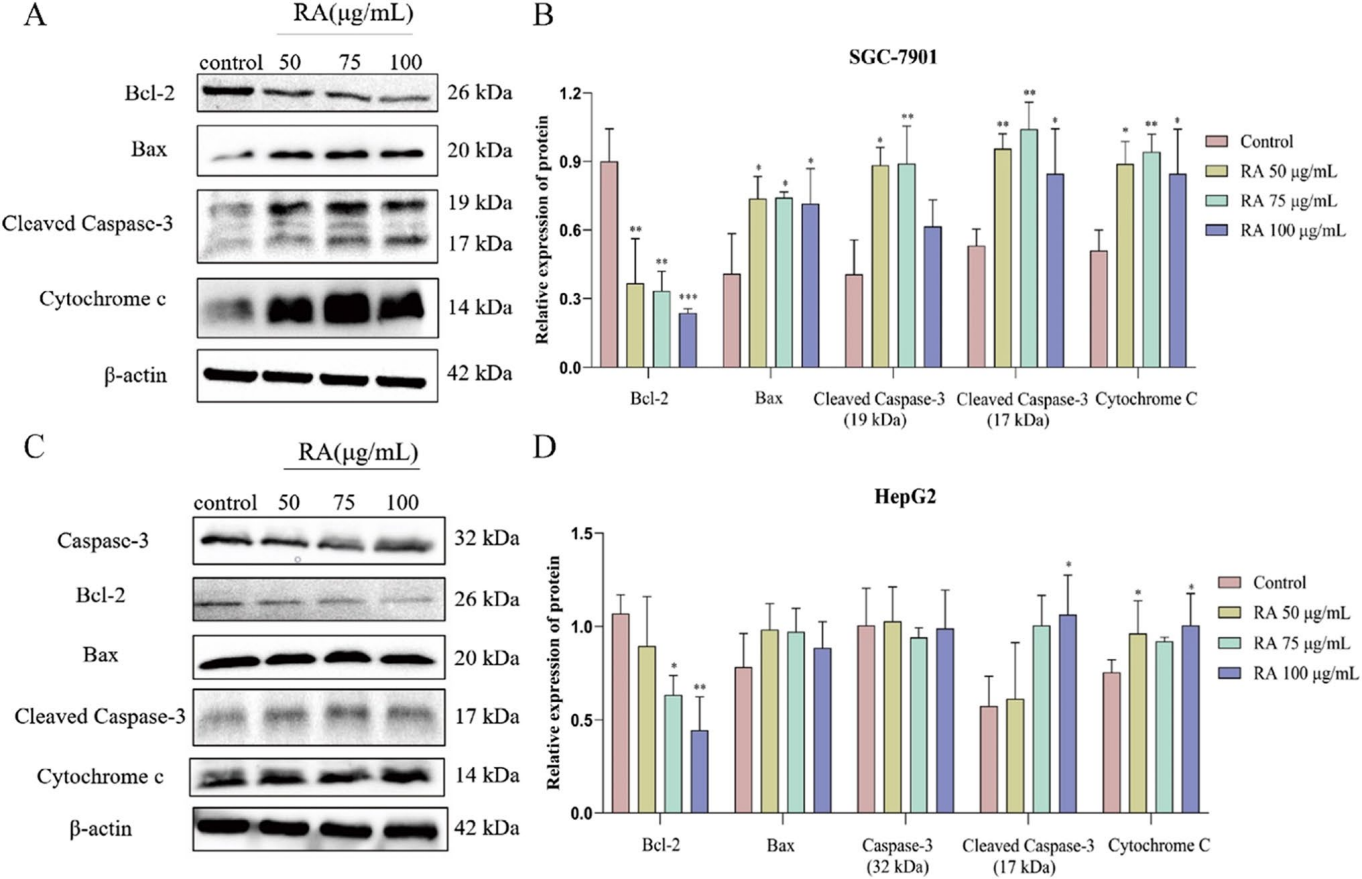
**A** SGC apoptosis related protein band. **B** and **D** Western blot analysis of protein expressions of Bcl-2, Bax, caspase3, cleaved caspase 3 (17 kDa and 19 kDa), and Cyt C (*n* = 3). **C** HepG2 apoptosis related protein band. The data are presented as the mean ± SD from three independent experiments, *P < 0.05, **P < 0.01, ***P < 0.001 vs control. Cells not treated were used as control

To explore the mechanism of RA-induced apoptosis of SGC-7901 and HepG2 cells, western blotting was performed to detect the expression of apoptosis-related proteins. As shown in Fig. 6A and C, compared with control group, the expression levels of Bax, cleaved caspase-3, and cytochrome C protein in SGC-7901 and HepG2 cells were significantly increased after treatment with RA, while Bcl-2 decreased. As shown in Fig. 6B and D, the expression levels of apoptosis-related proteins in these two cells were slightly different. Overall, these data indicated that RA induced apoptosis in both SGC-7901 and HepG2 cells.

## Discussion

RA can be found in Boraginaceae and Lamiaceae families. It mainly exists in the leaves of *Rosmarinus officinalis*, from which it can be easily isolated. It is also present in peppermint, lemon balm, oregano, sage, and thyme (Sengelen and Onay-Ucar 2018; Juskowiak et al. 2018). RA has been isolated from RF for the first time. According to the literature, RA possesses a variety of pharmacological activities, mainly focusing on antioxidant (Yang et al. 2013) and anti-inflammatory (Chu et al. 2012) effects. In addition, RA has the effect of anti-tumor activity against a variety of cancer cells in vitro, including breast cancer, prostate cancer, and colon cancer. However, the specific mechanism of RA for inducing apoptosis of gastric and liver cancers is unclear. This study mainly discussed and evaluated the mechanism and efficacy of RA-inducing apoptosis of SGC-7901 and HepG2 cells in vitro.

We first detected the viability of the SGC-7901 and HepG2 cells, and the results showed that the cell decreased with an increase in RA concentration, which was consistent with that of the literature (Jin et al. 2020). Subsequently, we observed the effect of RA on the morphology of cells by an inverted fluorescence microscope and found that the morphology of cells in the treatment group showed different degrees of apoptosis. These results suggest that, with an increasing RA concentration, the number of dead cells floating in the upper layer of the culture medium also increases, and the size of the cells shrinks and gradually contracts into a circle and separated from the surrounding cells to gradually form apoptotic bodies. The appearance of the apoptotic bodies is generally considered to be one of the characteristics of apoptosis (Moujalled et al. 2021). On the other hand, one reason why tumors are difficult to cure completely is because of their migratory nature (Sivori et al. 2021). In this regard, we investigated whether RA could reduce the migration of gastric and liver cancer cells. The results revealed that RA inhibited the migration of tumor cells in a dose-dependent manner, showing the strongest inhibitory effect on SGC-7901 of gastric cancer cells.

Apoptosis is considered to be a form of programmed cell death (Moujalled et al. 2021). In the aforementioned experiments, as apoptotic bodies appeared after RA treatment, we speculated that RA possibly affected cell viability by inducing apoptosis. The apoptosis rate of SGC-7901 and HepG2 cells was measured by flow cytometry to reveal that the apoptosis rate of the two cells increased in a dose-dependent manner with the concentration of RA, showing an increase in the administration concentration of the two cells. We also noted an increasing trend in the proportion of late apoptosis, which suggested that RA affects cell viability by inducing apoptosis. This report is similar to previous reports in the literature (Hong et al. 2021) indicating that RA has an apoptosis-inducing effect on tumor cells.

Apoptosis and cell cycle have always been necessary experimental items to detect the mechanism and strength of drugs on tumor cells (Ren et al. 2022). The cell cycle represents a series of events from the growth until the death of cells. The entire process consists of two phases, namely, the intercellular phase and the cell division phase. The intercellular phase can be specifically categorized into the G0/G1, S, and G2 phases (Sablowski and Carnier Dornelas 2014). In recent years, during the development of anti-tumor drugs, these different stages of the cell cycle have become the key targets for tumor therapy. The G0/G1 phase, also known as the early stage of DNA synthesis, mainly synthesizes RNA and protein to prepare for DNA replication. The main feature of this stage is the increase of various enzyme concentrations related to DNA replication in the cell, as well as an increase in the mitochondria, chloroplasts, and ribosomes. The S phase is the DNA synthesis phase, in which DNA starts to replicate, and its number is between 2 and 4N, and histones and DNA are synthesized to form chromatin. The G2 phase is the late stage of DNA synthesis, in which DNA synthesis is terminated, and RNA and protein synthesis in the cells begin to prepare for cell division (Jimenez-Sanchez 2017). During the entire cell cycle, the next stage can only progress after the completion of the relevant biochemical reactions in the previous one. All events that could affect the entry into the next stage, including inside and outside the body, during the transition between the upper and lower periods, then this transition period, which reversibly arrests the cell cycle in the phase affected by the corresponding event, is called the checkpoint, and the period in which the corresponding impact stops is called cell cycle arrest (Ratti et al. 2018). Based on this finding, we investigated the effect of RA on the cell cycle distribution of SGC-7901 and HepG2 cells to explore the mechanism of RA-induced apoptosis in SGC-7901 and HepG2 cells. The effect of RA on the cell cycle of these two tumor cells was examined by flow cytometry, and the results revealed that, in SGC-7901 cells, RA could dose-dependently induce the G0/G1 phase arrest in the cell cycle, thereby reducing the proportion of the S phase, because RA affects the function of mitochondria in the G0/G1 phase, resulting in the production of insufficient raw materials for DNA synthesis and blocking in the G0/G1 phase, which further confirms that RA-induced apoptosis of SGC-7901 cells may occur through the mitochondrial apoptosis pathway. In HepG2 cells, RA also blocked the cell cycle in the S phase in a dose-dependent manner, indicating that RA may induce cell damage or mutation during its DNA synthesis to block the cell cycle in the S phase. This observation is slightly different from that of RA-induced colon cancer cell cycle arrest reported in the literature. The past reports support that RA arrests the HepG2 cell cycle in the G0/G1 phase (Han et al. 2018). We had a different observation in the present study possibly because of the location of the checkpoints in the cell cycle. There are two key checkpoints in the cell cycle, referring to the positions in the G1/S and G2/M phases, respectively, and the G1 and S phases in the early and mid-stage processes of DNA synthesis. In these two processes of DNA synthesis, as long as some factor affects DNA synthesis, the phenomenon of the G1 or S phase arrest will appear. However, in general, RA can induce apoptosis by blocking the G0/G1 phase of SGC-7901 cells and the S phase of HepG2 cells.

Apoptosis can mainly be divided into endogenous and exogenous pathways. Among the endogenous pathways, mitochondria-mediated apoptosis is an important way to induce tumor cell apoptosis (Estaquier et al. 2012). Generally, when tumor cells are affected by internal or external stimuli, such as DNA damage and oncogene activation, the mitochondrial apoptosis pathway can be activated, and the tumor cells go through the following processes again. Firstly, the Bcl-2 family proteins control the mitochondrial membrane permeability by regulating the mitochondrial membrane potential. In normal cells, the anti-apoptotic proteins Bcl-2 and Bcl-xL form heterodimers with the pro-apoptotic proteins Bax and Bak to maintain the integrity of the mitochondrial membrane. The release of Bax/Bak, in turn, releases the apoptotic factor cytochrome C, which then changes the permeability of the mitochondrial membranes (Kim et al. 2007). Then, due to the change in the mitochondrial membrane permeability, cytochrome C is released into the cytoplasm, where it combines with the apoptosis activator Apaf-1 to form a polymer, which then activates the apoptosis protein caspase-3, ultimately resulting in the activation of the caspase cascade, leading to apoptosis (Yang et al. 2014). Our western blotting results suggested that RA could dose-dependently inhibit the expression of anti-apoptotic protein Bcl-2, enhance the expression of pro-apoptotic proteins Bax and cytochrome C, and promote the activated expression of caspase-3. These results suggested that RA induced the apoptosis of SGC-7901 and HepG2 cells through the mitochondrial apoptosis pathway.

Generally, the abovementioned data indicate the effect of RA on the induction of apoptosis in gastric cancer SGC-7901 cells and liver cancer HepG2 cells, suggesting the action mechanism behind the same, which can serve as a scientific basis for the treatment of RA on tumors and contributes to the knowledge on the anti-tumor activity of RF. The action mechanism revealed herein provides directions for the further development and utilization of RF. In addition, since RA is a natural phenolic acid compound, its antioxidant properties may be related to the induction of tumor cell apoptosis, because when cells are stimulated by oxidative stress, a large number of reactive oxygen species are produced in the mitochondria, and the amount of reactive oxygen species production also changes the mitochondrial membrane permeability and triggers the mitochondrial apoptotic pathway (Rizwan et al. 2020). This experimental result did not suggest any association of its antioxidant activity with the induction of apoptosis. Therefore, in the follow-up analyses, we explored the relationship between RA antioxidant activity and mitochondrial apoptosis through an in vivo experiment. We also explored the anti-tumor action forms and the pathways of RA in vivo, providing more methods for tumor treatment.

## Conclusion

**Fig. 7:**
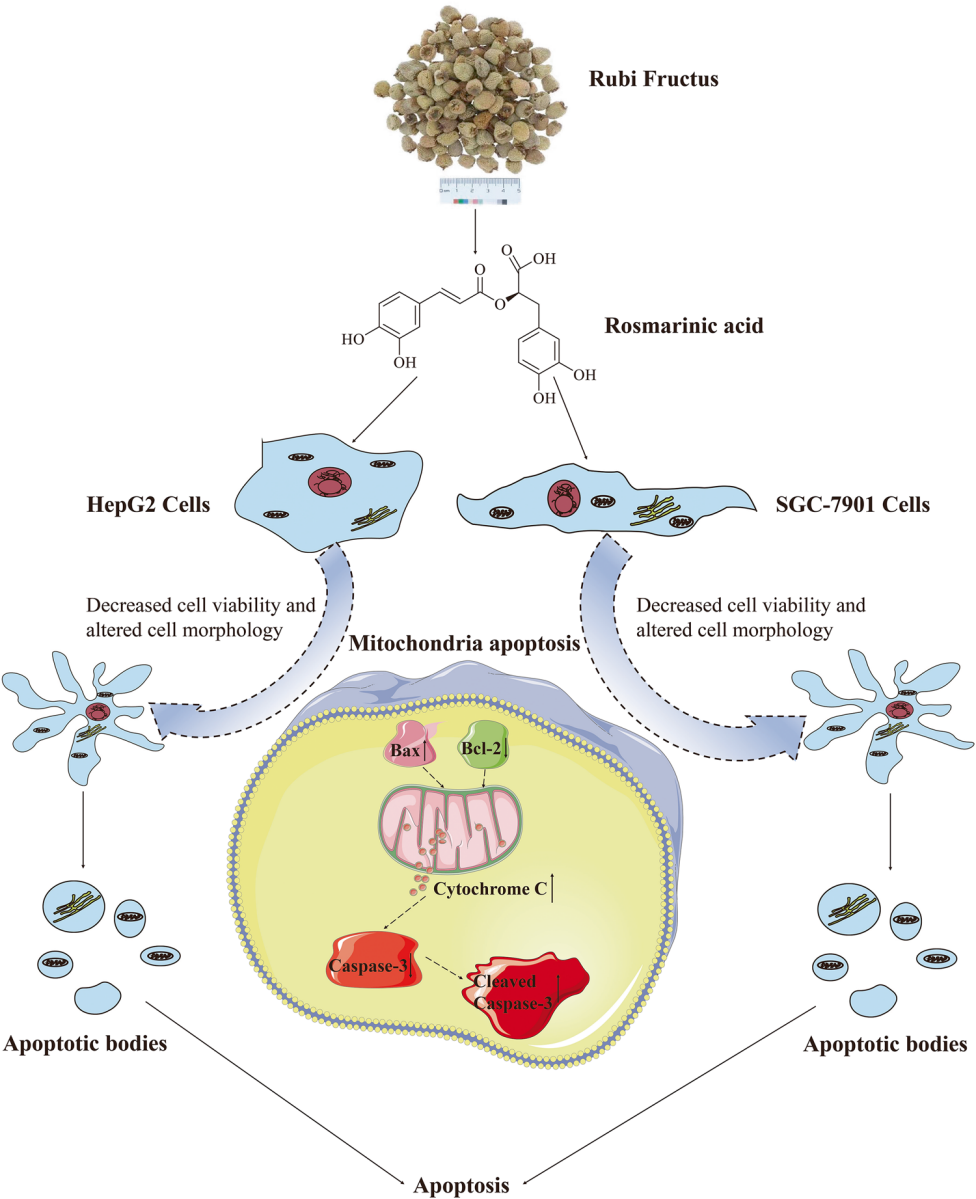
Mechanism of RA-induced apoptosis in SGC-7901 and HepG2 cells

In this study, we performed extraction, separation, and then identification of the active components in RF to isolate RA for the first time. In vitro experiments and western blotting results showed that RA could inhibit SGC-7901 and HepG2 cells through the mitochondrial apoptosis pathway (Fig. 7). This study also found the mechanism of RA-inducing apoptosis of SGC-7901 and HepG2 cells, which further enriches the material basis of RF in anti-tumor properties.

### Supplementary Information

Below is the link to the electronic supplementary material.

Supplementary file1 (ZIP 25020 kb)

## Data Availability

The data used to support the findings of this study are included within the article.
