# Supplementary material for: Rosmarinic acid, the active component of Rubi Fructus, induces apoptosis of SGC-7901 and HepG2 cells through mitochondrial pathway and exerts anti-tumor effect
Source: Naunyn Schmiedebergs Arch Pharmacol. 2023 Jun 20;396(12):3743–55. doi: 10.1007/s00210-023-02552-z (PMC10643355; doi:10.1007/s00210-023-02552-z)
Supplement: Supplementary file 1 — Supplementary file1 (ZIP 25020 kb) [file 210_2023_2552_MOESM1_ESM.zip › Supplementary Material.docx]

**Rosmarinic acid, the active component of Rubi Fructus，induce apoptosis of SGC-7901 and HepG2 cells through mitochondrial pathway and exert anti-tumor effect**

Naunyn-Schmiedeberg's Archives of Pharmacology

Changlun Chen ^†^, Yilin Liu ^†^, Yi Shen, Lili Zhu, Lumeng Yao, Xingxing Wang, Anna Zhang, Jiao Li, Jianjun Wu^*^, Luping Qin^**^

College of Pharmaceutical Sciences, Zhejiang Chinese Medical University, Hangzhou, 311402, China

Correspondence should be addressed to Luping Qin; [lpqin@zcmu.edu.cn](mailto:lpqin@zcmu.edu.cn) and Jianjun Wu; [wjjpharmacy@163.com](mailto:wjjpharmacy@163.com)

† These authors contributed equally to this work.

Fig. S1. Rosmarinic acid ^1^H NMR, ^13^C NMR, and 2D-NMR dates: 2D-HSQC , 2D-COSY and 2D-HMBC

^1^H NMR

^13^C NMR and DEPT (135)

2D-HSQC , 2D-COSY and 2D-HMBC
