# Supplementary material for: Rosmarinic acid, the active component of Rubi Fructus, induces apoptosis of SGC-7901 and HepG2 cells through mitochondrial pathway and exerts anti-tumor effect
Source: Naunyn Schmiedebergs Arch Pharmacol. 2023 Jun 20;396(12):3743–55. doi: 10.1007/s00210-023-02552-z (PMC10643355; doi:10.1007/s00210-023-02552-z)

HepG2 cells

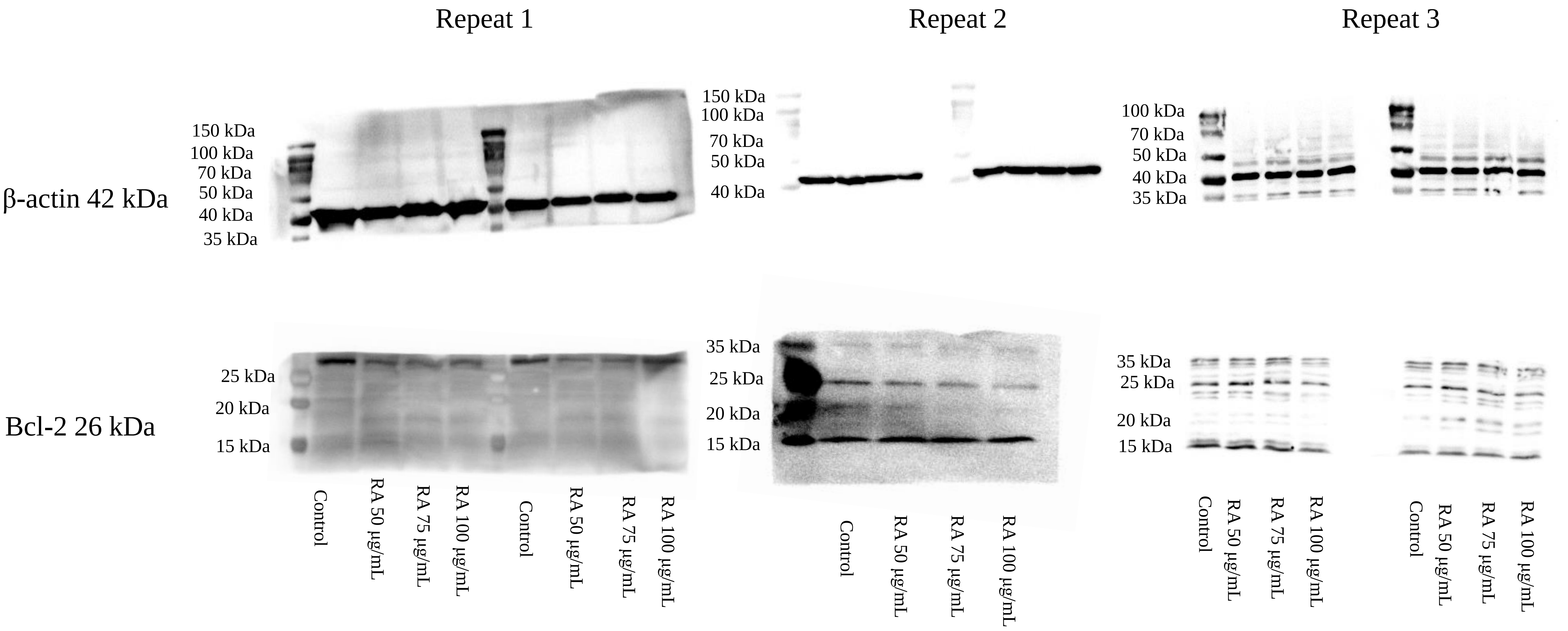
Repeat 1
Repeat 2
Repeat 3
β-actin 42 kDa
Bcl-2 26 kDa
150 kDa
100 kDa
70 kDa
50 kDa
40 kDa
35 kDa
25 kDa
20 kDa
15 kDa
Control
RA 50 μg/mL
RA 75 μg/mL
RA 100 μg/mL

HepG2 cells

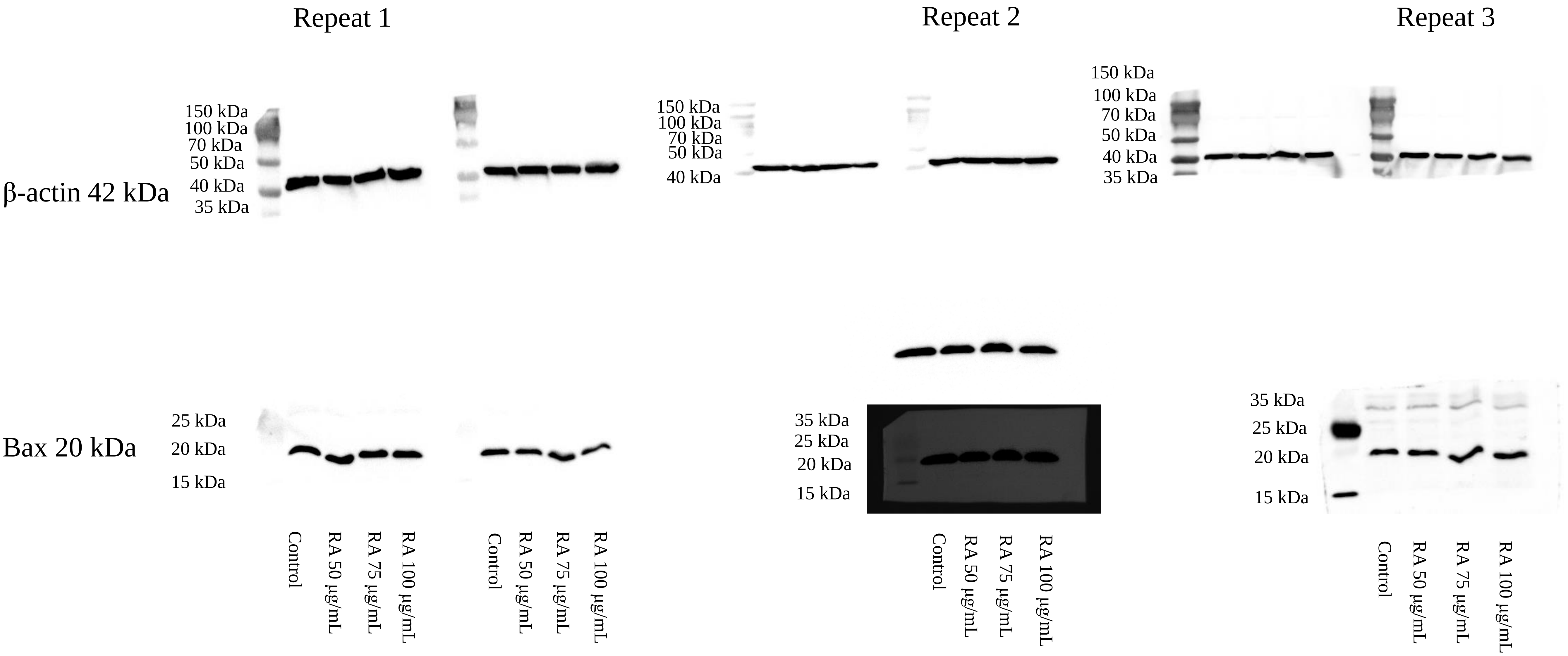
Repeat 1
Repeat 2
Repeat 3
β-actin 42 kDa
150 kDa
100 kDa
70 kDa
50 kDa
40 kDa
35 kDa
Bax 20 kDa
25 kDa
20 kDa
15 kDa
Control
RA 50 µg/mL
RA 75 µg/mL
RA 100 µg/mL

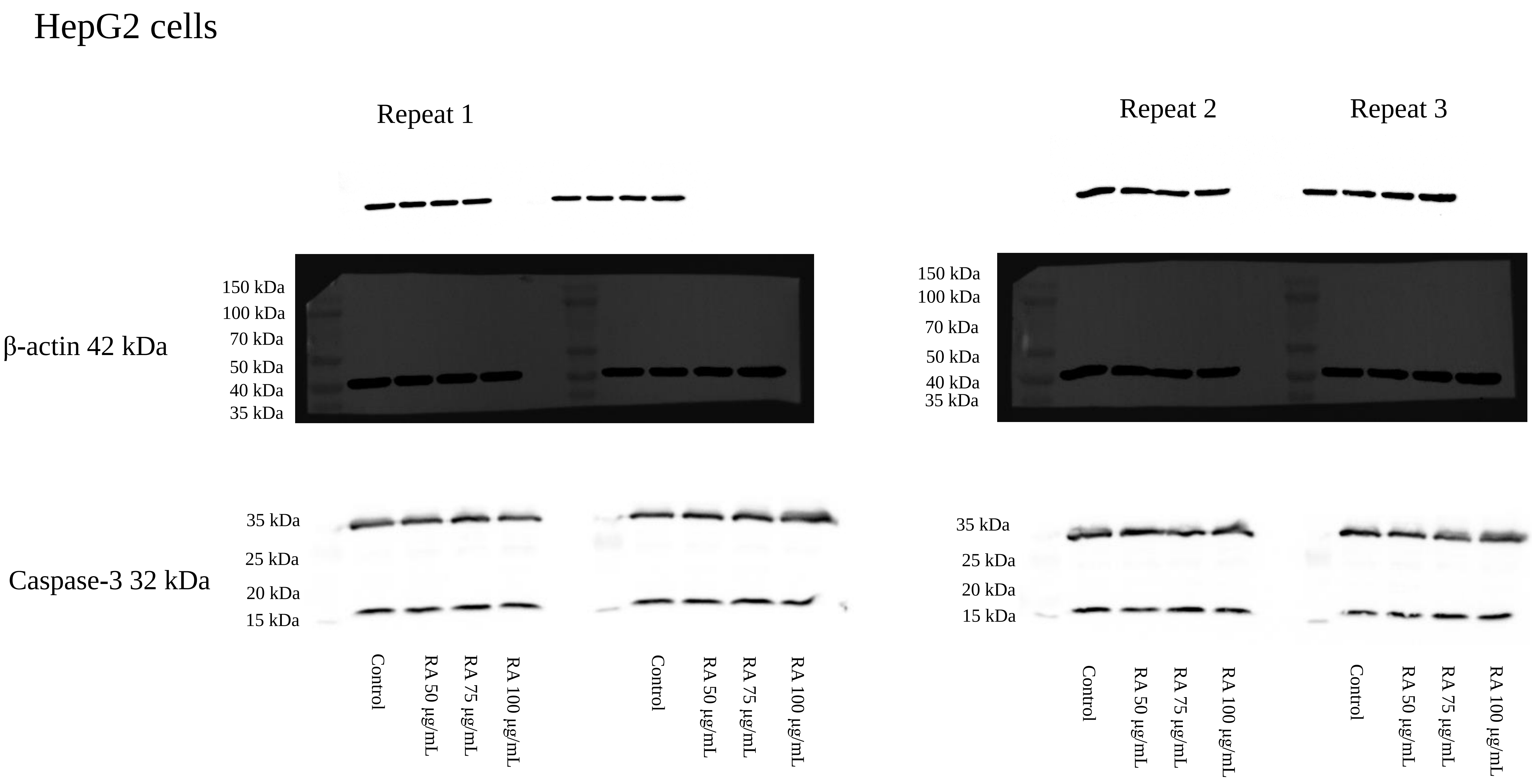
HepG2 cells
Repeat 1
Repeat 2
Repeat 3
β-actin 42 kDa
150 kDa
100 kDa
70 kDa
50 kDa
40 kDa
35 kDa
Caspase-3 32 kDa
35 kDa
25 kDa
20 kDa
15 kDa
Control
RA 50 µg/mL
RA 75 µg/mL
RA 100 µg/mL

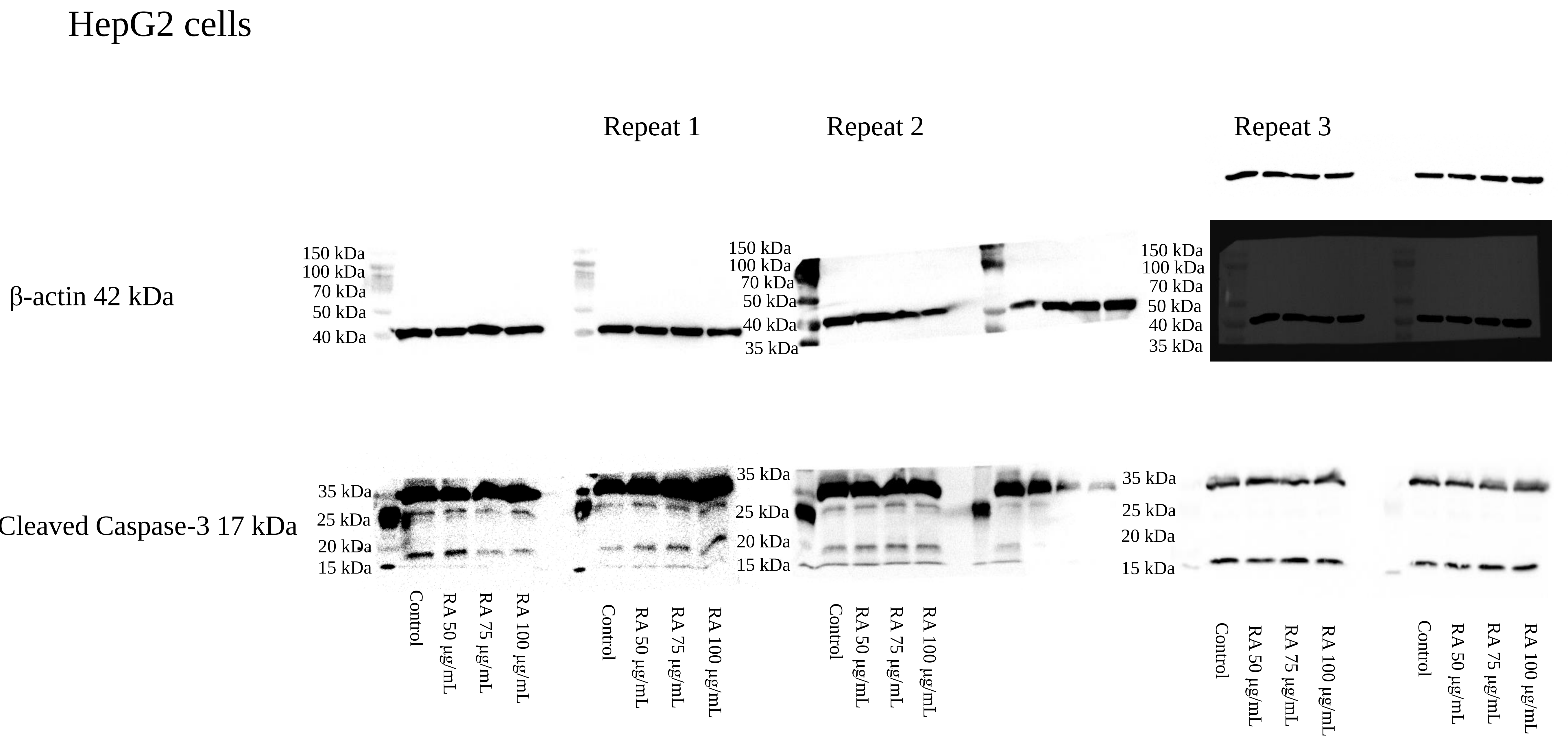
HepG2 cells
Repeat 1
Repeat 2
Repeat 3
β-actin 42 kDa
Cleaved Caspase-3 17 kDa
150 kDa
100 kDa
70 kDa
50 kDa
40 kDa
35 kDa
25 kDa
20 kDa
15 kDa
Control
RA 50 µg/mL
RA 75 µg/mL
RA 100 µg/mL

HepG2 cells

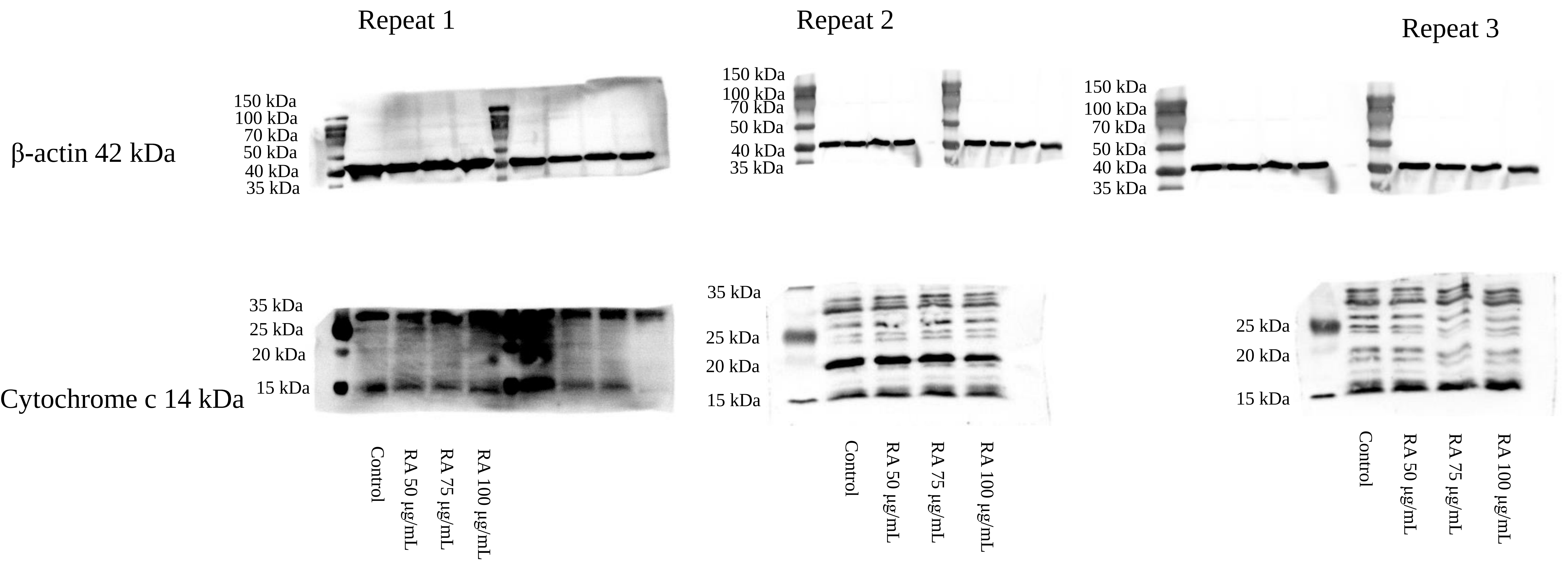

Repeat 1
Repeat 2
Repeat 3
β-actin 42 kDa
Cytochrome c 14 kDa
150 kDa
100 kDa
70 kDa
50 kDa
40 kDa
35 kDa
25 kDa
20 kDa
15 kDa
Control
RA 50 µg/mL
RA 75 µg/mL
RA 100 µg/mL

SGC-7901 cells

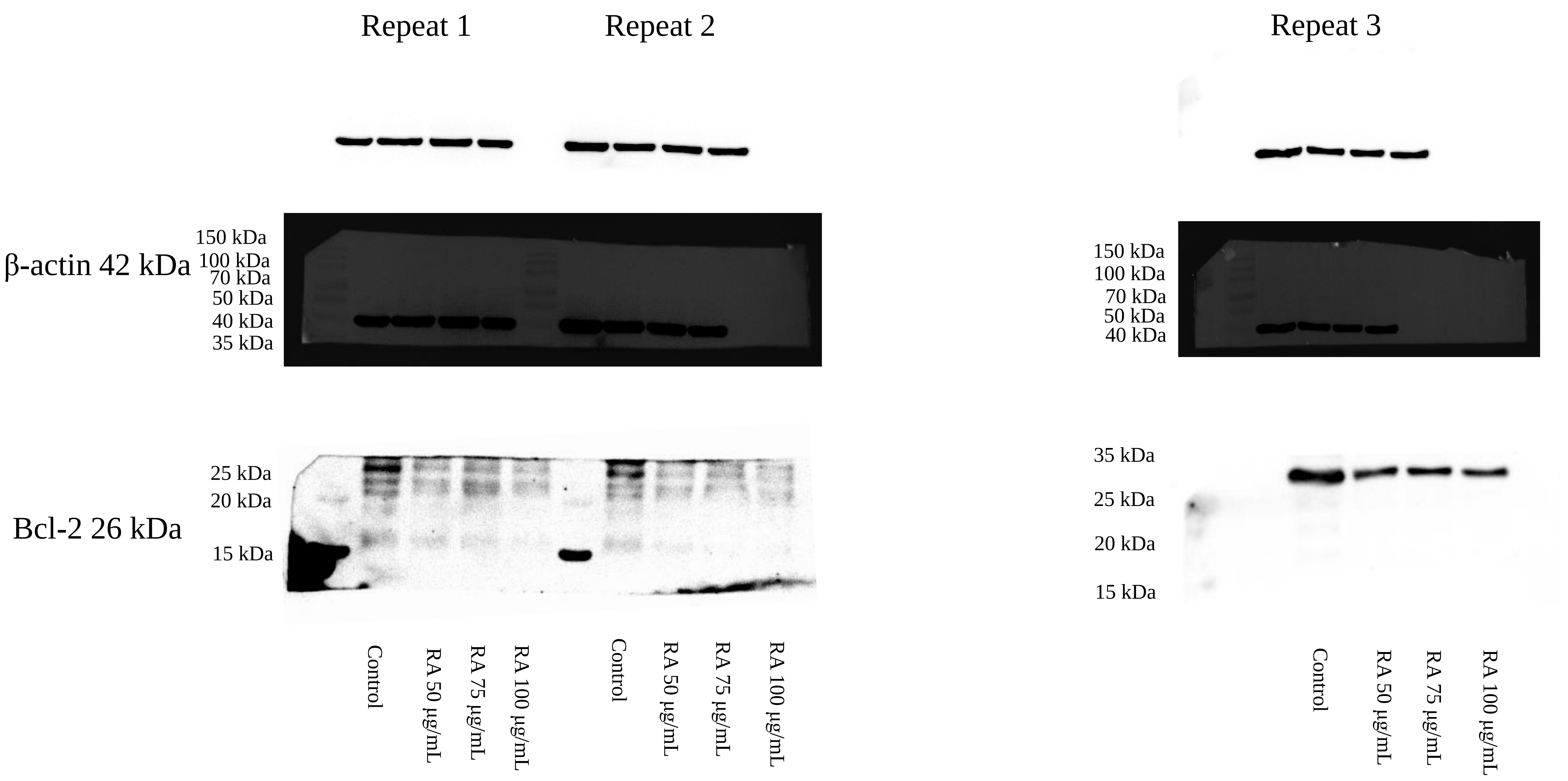

SGC-7901 cells

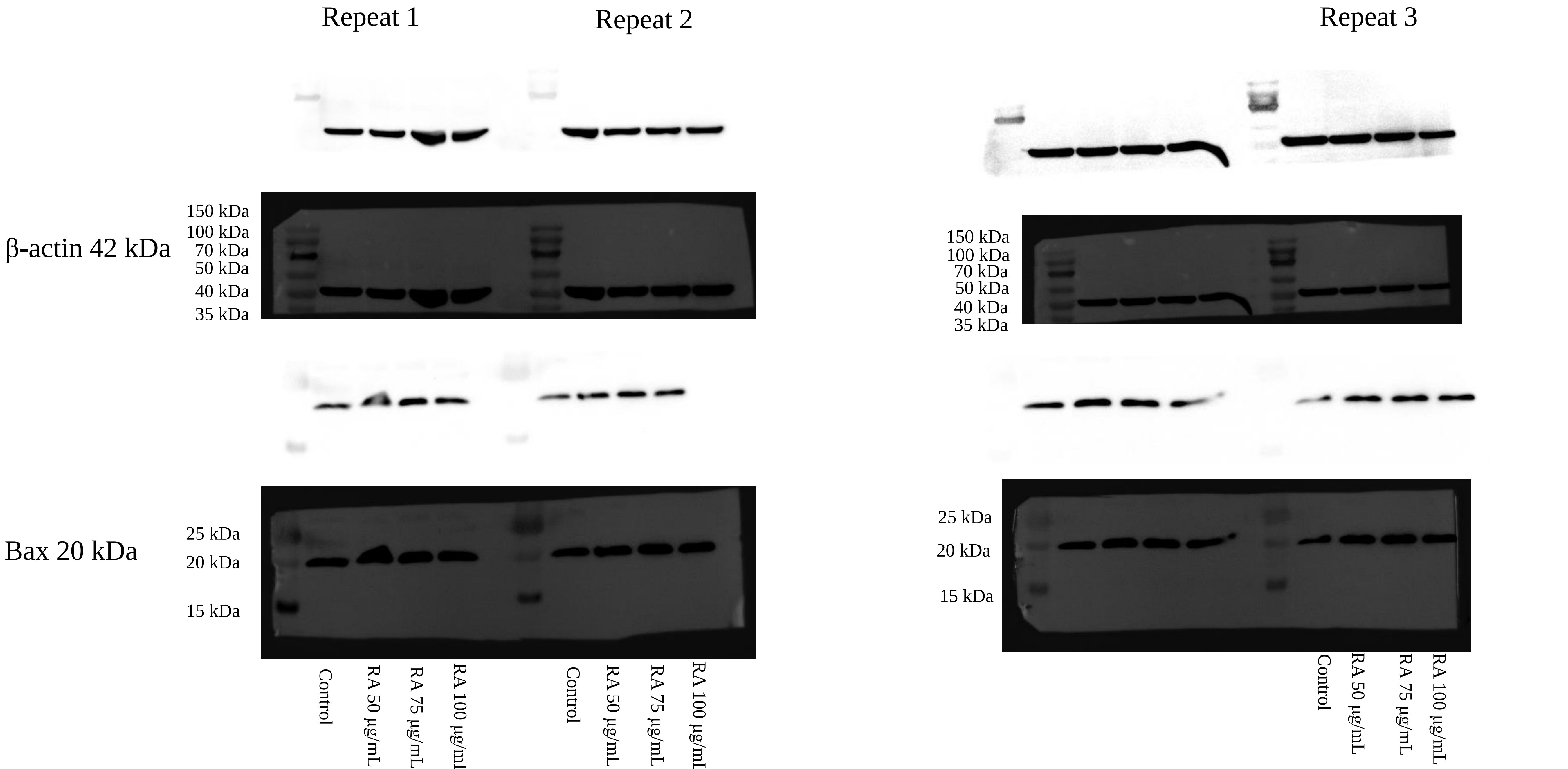

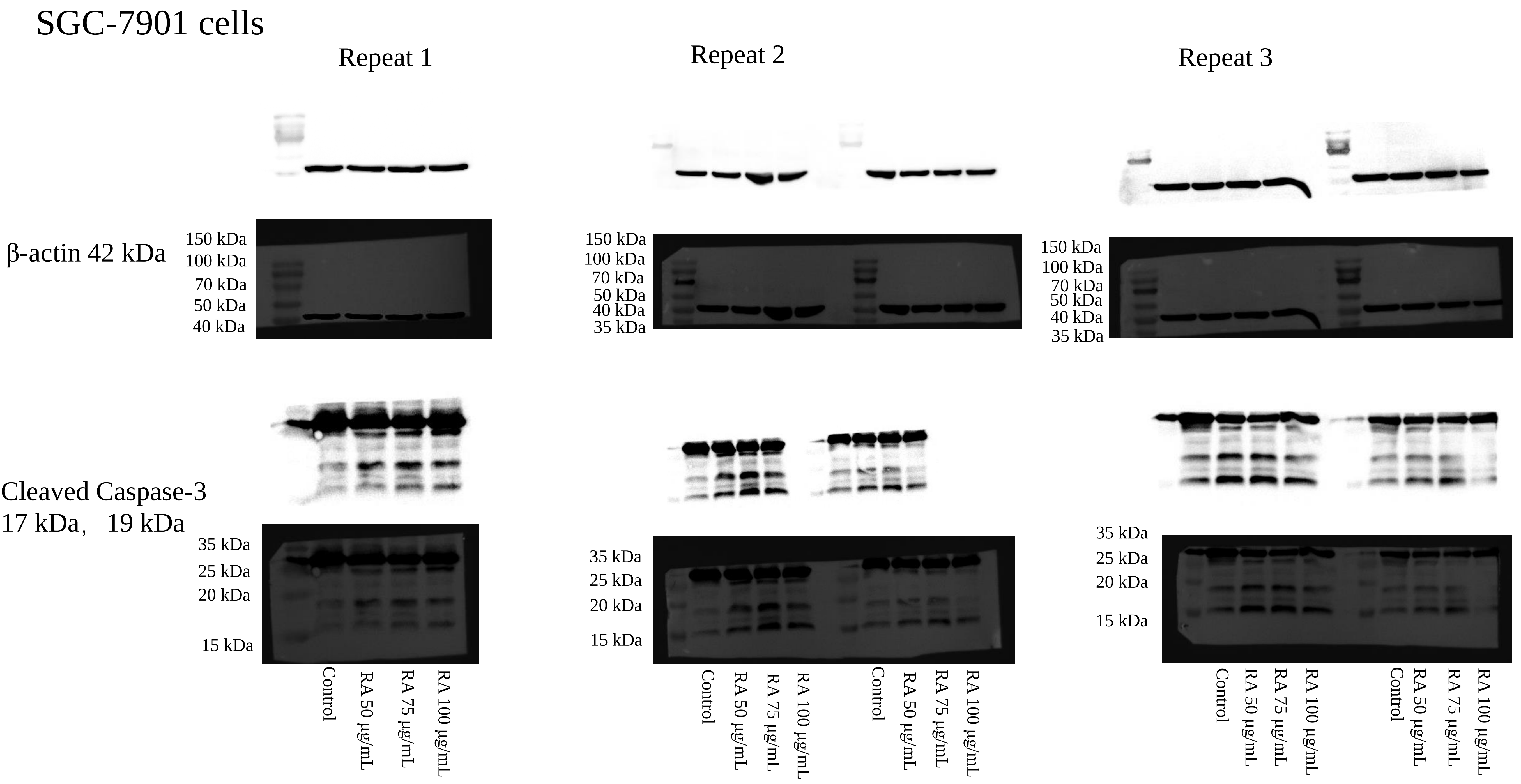

SGC-7901 cells
Repeat 1
Repeat 2
Repeat 3
β-actin 42 kDa
150 kDa
100 kDa
70 kDa
50 kDa
40 kDa
35 kDa
Cleaved Caspase-3
17 kDa， 19 kDa
35 kDa
25 kDa
20 kDa
15 kDa
Control
RA 50 μg/mL
RA 75 μg/mL
RA 100 μg/mL

SGC-7901 cells

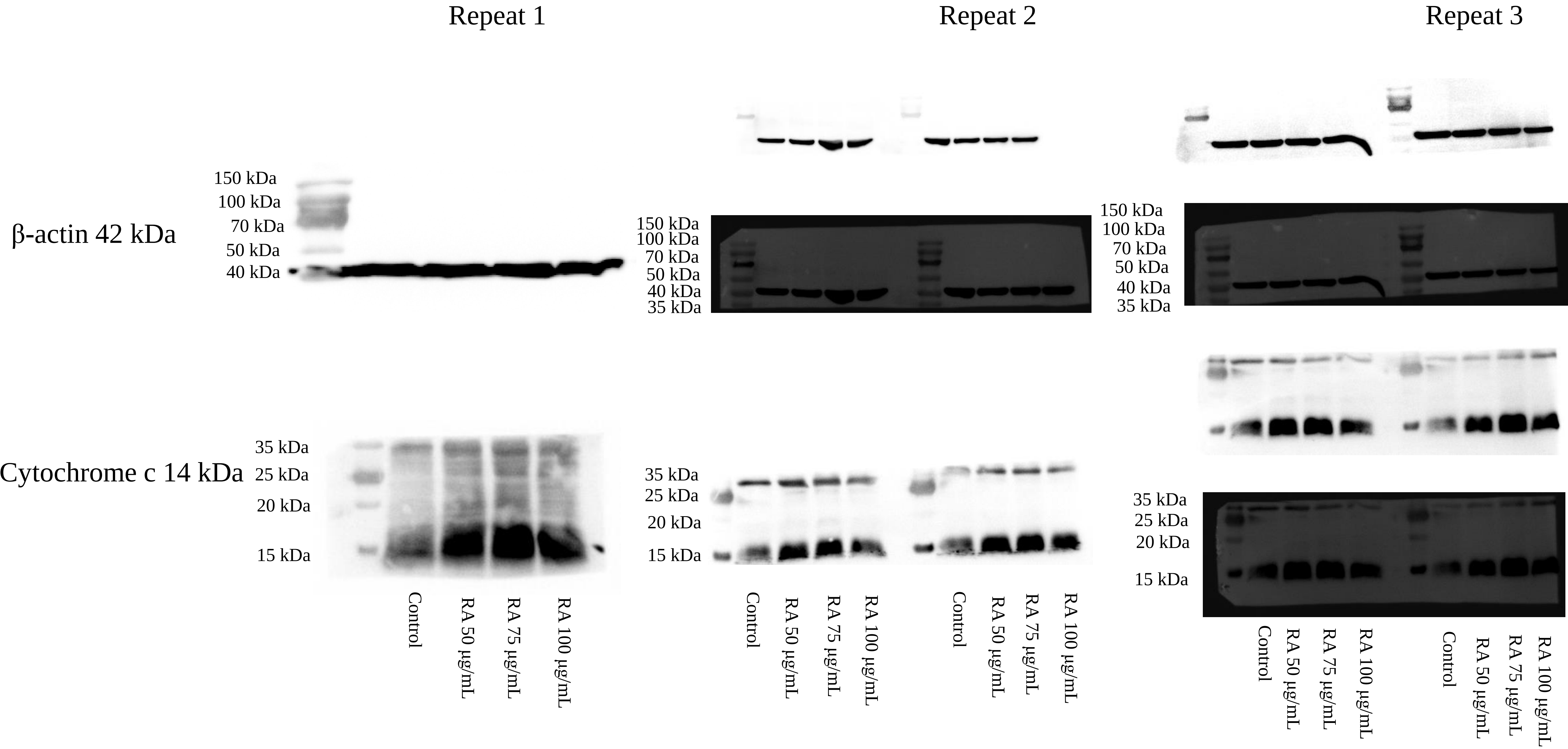

Supplement: Supplementary file 1 — Supplementary file1 (ZIP 25020 kb) [file 210_2023_2552_MOESM1_ESM.zip › WB original picture.pdf]
