# Supplementary figures and images for: Rosmarinic acid, the active component of Rubi Fructus, induces apoptosis of SGC-7901 and HepG2 cells through mitochondrial pathway and exerts anti-tumor effect
Source: Naunyn Schmiedebergs Arch Pharmacol. 2023 Jun 20;396(12):3743–55. doi: 10.1007/s00210-023-02552-z (PMC10643355; doi:10.1007/s00210-023-02552-z)

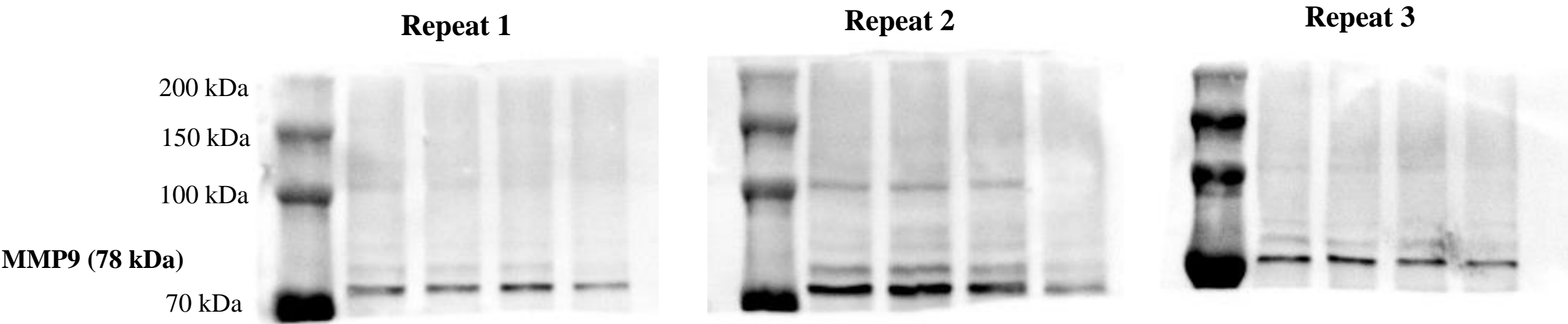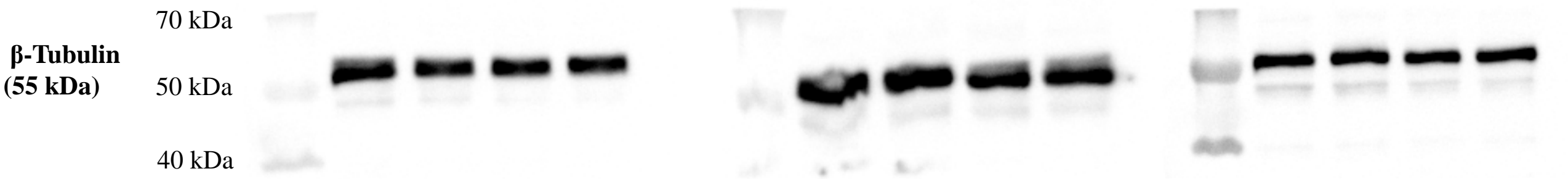

**HepG2 cells**

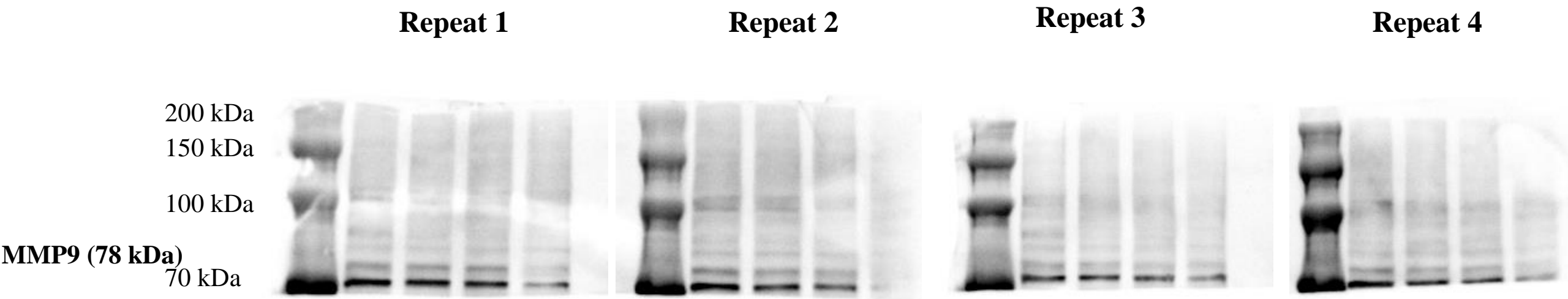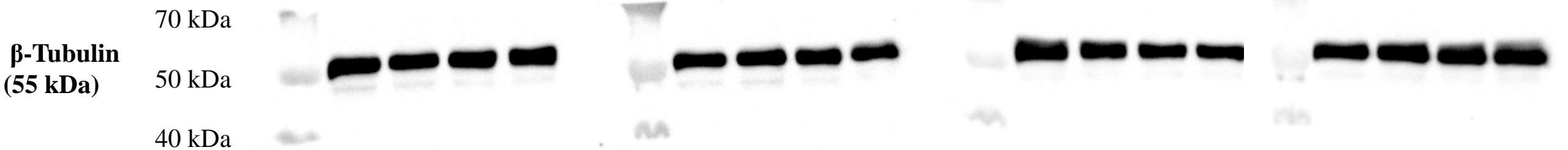

**SGC-7901 cells**

Supplement: Supplementary file 1 — Supplementary file1 (ZIP 25020 kb) [file 210_2023_2552_MOESM1_ESM.zip › MMP9 WB original picture.pdf]
